# Intensification of Biophenols Extraction Yield from Olive Pomace Using Innovative Green Technologies

**DOI:** 10.3390/biom13010065

**Published:** 2022-12-29

**Authors:** Yosra Belghith, Imen Kallel, Maxence Rosa, Panagiotis Stathopoulos, Leandros A. Skaltsounis, Noureddine Allouche, Farid Chemat, Valérie Tomao

**Affiliations:** 1 Natural Substances Team, Laboratory of Organic Chemistry LR17ES08, Faculty of Sciences of Sfax, University of Sfax, P.B. 1171, Sfax 3000, Tunisia; 2 MicroNut Team, INRA, UMR408, Université d’Avignon, F-84000 Avignon, France; 3 Laboratory of Toxicology-Environmental Microbiology and Health Research (LR17ES06), Faculty of Sciences of Sfax, University of Sfax, Sfax 3000, Tunisia; 4 Division of Pharmacognosy and Natural Products Chemistry, Department of Pharmacy, NKUA, 15771 Athens, Greece; 5 GREEN Team, INRA, UMR408, Université d’Avignon, F-84000 Avignon, France

**Keywords:** olive pomace, polyphenol extraction, COSMO-RS software, accelerated solvent extraction, ultrasound, bead milling, microwave

## Abstract

Olive pomace is the main by-product generated by the olive oil production process. Although toxic to the environment, olive pomace is an important source of natural antioxidants due to its high content of phenolic compounds. The aim of the current study is to maximize the extraction yields of the main phenolic compounds present in olive pomace using innovative green technologies. For this purpose, the present work is divided into two parts. The first part is based on a solubility study of targeted phenolic compounds in various ethanol/water ratios at two different temperatures (20 °C and 50 °C). A computational prediction using COSMO-RS software was applied for the calculation of eventual solubility, which was subsequently confirmed by practical experiments. The determination of the optimal extraction conditions of solvent ratio (EtOH/H_2_O) (60:40 *v*/*v*) and temperature (50 °C) led to the second part of the work, which concerns the intensification of extraction yields. Furthermore, various green extractions using innovative technologies, including accelerated solvent extraction (ASE), ultrasound with its both system (probe (UAE-P) and bath (UAE-B)), bead milling (BM) and microwave (MAE), were carried out and then compared to conventional maceration (CM). Results showed that ASE was the most effective method for extracting phenolic compounds from dried olive pomace powder (5.3 milligrams of tyrosol equivalent (TE) per gram of dried olive pomace powder (DOP)) compared to CM (3.8 mg TE/g DOP).

## 1. Introduction

Olive oil production is the second most important agriculture sector in Europe, and its output tends to increase. As a result, large volumes of waste from olive oil production are released into the environment, contributing to an excessive nutrient load in local ecosystems with ecological risks [1]. The quantity and characteristics of the generated waste depend on the processing conditions, the agricultural specificities and the season. The process of three-phase systems generates a solid by-product and waste waters [2] while the process of two phases systems produces only a wet olive pomace containing vegetation water with solid olive particles (peel, stone and pulp) called “olive pomace”. The latter system generates lower volumes of by-products but the effluents produced also have phytotoxic and antimicrobial properties, mainly due to the presence of phenolic compounds [3]. Only 2% of the total phenolic content (TPC) persists in olive oil during the extraction process, and the main fraction of TPC (about 98%) migrates to the resulting waste [4,5]. Nevertheless, olive pomace may also be considered a valuable and inexpensive source of bioactive compounds such as phenolic compounds, which are recognized for their potential health benefits [6,7,8,9,10,11,12,13].

The structural diversity of phenolic compounds, ranging from a simple phenolic molecule to complex high molecular weight polymers, promotes their potential to act as defense mechanisms against biotic and abiotic stress. Therefore, the recovery of these phenolic compounds represents an important objective of reducing the environmental impact of olive pomace and obtaining high added-value extracts. To extract phenols, ethanol/water mixtures have been widely accepted as a conventional solvent from different plant matrices (Table 1).

According to Lim et al. [17], Nawaz et al. (2006) [18], and Shi et al. [20], the ethanol–water binary solvent system was the best extraction solvent of phenolic compounds from mango seed kernel, grape seeds and grape seeds meal, respectively. Barros et al. [16] also reported that extraction yields of antioxidant molecules were improved by 12% while using ASE and ethanol/water mixtures (50% and 70%) compared to CM. In another study by Durling et al. [14], the optimal hydroalcoholic solvent ratio was shown to be between 55% and 75% of ethanol. Under specific conditions, this mixture of extraction solvent was able to recover 55% of rosmarinic acid, 75% of carnosic acid, and 42% of the essential oil from dried sage. On the same principle, Čepo et al. [15] showed that 60% of ethanol was the best solvent percentage for phenolic compounds extraction from olive pomace and in particular oleuropein (115.14 ± 0.19 mg/kg fresh olive pomace). In addition, the experiments of Wang et al. [19] showed that 25 min of UAE with hydroalcoholic solvent (64% ethanol) at 60 °C were the optimal extraction conditions to extract 3.12 mg GAE/g of phenolic compounds from wheat bran.

CM is generally associated with low extraction efficiency and may be improved by some green processes, which typically use less solvent and energy such as ASE [16,22], UAE in its two types (Probe (UAE-P) and bath (UAE-B)) [18,19,20,24] and MAE [25].

To our knowledge, there is no previous study dealing with the use of ASE and BM technologies and their comparison for the extraction of phenolic compounds from olive pomace.

**Figure 1:**
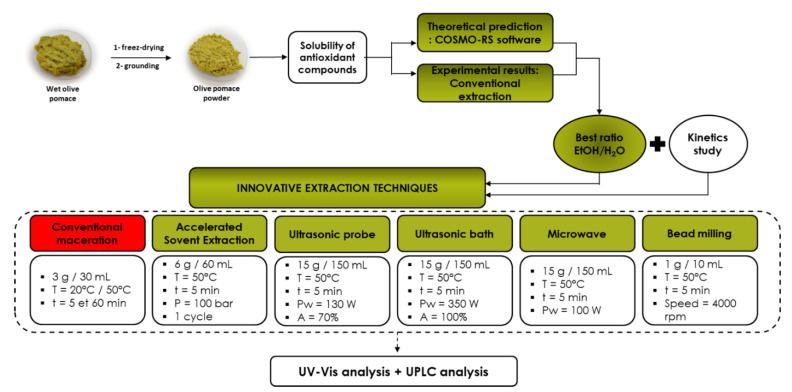
Global protocol for olive pomace extraction.

In the present investigation, the work focused on the optimization of the extraction conditions of phenolic compounds from olive pomace (Figure 1) by maceration at two different temperatures (20 °C and 50 °C) based on COSMO-RS software results. Subsequently, studies were conducted to improve the extraction rate using innovative green technologies with the optimal extraction conditions obtained by CM.

## 2. Materials and Methods

### 2.1. Raw Material

Olive pomace was collected from *Aglandau* olives in October 2020 according to organic farming rules in a two-phase olive oil mill (Moulin Castelas, Baux-de-Provence, France). Fresh pomace was immediately frozen and stored at −20 °C. Before analyses, the olive pomace was freeze-dried by a freeze-dryer (Cryotec cosmos-80, Saint-Gely-du-Fes, France) and then finely ground with a grinder (Qlive 870873, Villeneuve-d’Ascq, France). The moisture content of pomace powder was measured with a humidimeter (Ohaus MB35, Parsippany, NJ, USA) to present the results on a dry matter basis.

### 2.2. Chemicals

Folin–Ciocalteu reagent, sodium carbonate, hydroxytyrosol, caffeic acid and p-coumaric acid were purchased from Sigma-Aldrich (Deisenhofer, Germany), tyrosol (4-hydroxyphenylethanol) was from TCI (Tokyo, Japan) and ferulic acid was obtained from Extrasynthèse (Genay, France). Ethanol absolute anhydrous (99.9% of purity) was from Carlo Erba reagents (Cornaredo, Italy), acetonitrile of UHPLC grade was from VWR International (Darmstadt, Germany) and deionized water (18 mΩ) was obtained from Milli-Q system (Millipore, Burlington, MA, USA).

### 2.3. Methods

#### 2.3.1. Computational Method: COSMO-RS Software

Klamt and his co-workers developed a conductor-like screening model for real solvent as a computational prediction method [26], which is based on a combination of quantum chemistry (COSMO) with statistical thermodynamics (RS), for solvent screening and molecular description, in order to visualize the mechanism of dissolution through the electrostatic interaction between the solvents and solutes.

The first step of the COSMO model consists in simulating the virtual conductor environment in which the studied molecules are embedded. Under a given environment, the molecule induces a polarization charge density on its surface. Therefore, the self-consistent quantum-computing algorithm will convert the molecule into the optimal state of energy in the conductor according to the geometry and electron density of the molecule. In the current study, the standard quantum chemical process for the COSMO-RS approach (triple zeta valence polarized basis set (TZPV)) was used.

The second step uses statistical thermodynamic calculations. The polarization charge density is used to quantify the interaction energy of pairwise interacting surface segments with regard to hydrogen and electrostatics bonding. On the surface of each molecule, the 3D distribution of polarization charges is converted into a surface composition function (s-profile). This s-profile provides detailed information about the distribution of molecular polarity. Then, the chemical potential of the surface segment (σ-potential) is calculated using the thermodynamics of molecular interaction based on the obtained s-profile.

In this work, the COSMOthermX program (version C30 release 13.01, Leverkusen, Germany) was used for all the calculations. Hydroalcoholic mixtures of EtOH/H_2_O with a variation of the ethanol percentage from 0 to 100% with an increment of 10% were modeled. Calculations were conducted at temperatures of 20 °C and 50 °C for each mixture. After an exhaustive literature review, solute selection was based on the major polyphenols detected in olive pomace [27].

The representatives of polyphenols were tyrosol, hydroxytyrosol, the two main glucoside-hydroxytyrosols (hydroxytyrosol-3-*O*-glucoside and hydroxytyrosol-1-*O*-glucoside), Oleuropein and Nuzhenide. Regarding the category of phenolic acids, gallic acid, vanilic acid, caffeic acid, *p*-coumaric acid, ferulic acid and *trans*-cinnamic acid were studied.

By implementing the COSMO model in COSMOtherm software (C30 1401, CosmothermX14, COSMOlogic GmbH &CO. KG, Leverkusen, Germany), the relative solubility of the main compounds of olive pomace under different ratios of EtOH/H_2_O can be calculated. The calculation is performed as follows:

$$\log_{10}\left(xj\right)=\log_{10}[\;\frac{exp\left(\mu_{j}^{pure}-\mu_{j}^{solvent}-\Delta G_{j,\;fusion}\right)}{RT}]$$

- *x_j_*: solubility of *j*;
- *μ_j_^pure^*: chemical potential of pure compound *j*;
- *μ_j_^solvent^*: chemical potential of j at infinite dilution;
- Δ*G_j,fusion_*: free energy of fusion of *j*.

**Figure 2:**
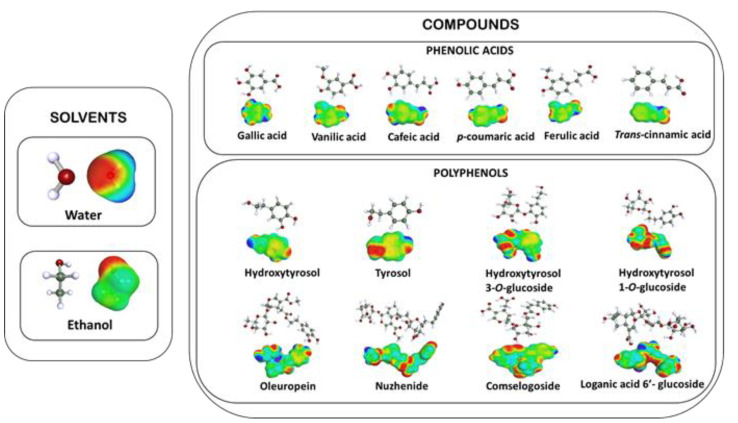
Molecular structures and σ-surfaces of the solvents and investigated solutes.

The relative solubility is always calculated at an infinite dilution. The logarithm of the best solubility is set to 0, and all other solvents are ranked relative to the best or reference solvent. The molecular structures and σ-surfaces of solvents and investigated solutes are presented in Figure 2.

#### 2.3.2. Conventional Extraction Method

First, 5 g of olive pomace powder was suspended in 50 mL of EtOH/H_2_O mixture under stirring at 300 rpm. The phenol concentration was monitored by UV/Vis spectroscopy for 3 h. The EtOH/H_2_O ratio tested was defined as a variation of the percentages of ethanol from 0 to 100% with 10% of increment at temperatures of 20 °C and 50 °C for each mixture. The extraction temperature was measured with an external sensor and regulated at 20 °C ± 2 °C then at 50 °C ± 2 °C. All experiments were performed in triplicate.

### 2.4. Intensification of Extraction Methods

All extractions were carried out over 5 min at 50 °C using a mixture of ethanol/water (60:40, *v*/*v*) with a ratio of sample/solvent equal to 1:10 (g/mL). After each extraction method, the filtered extracted solution underwent total phenolic content analyses; filtration was carried out using a 0.45 µm PTFE filter (Alltech Associates, Deerfield, IL, USA). A sample of each filtered extract was stored at −18 °C in the dark until UPLC analysis.

#### 2.4.1. Accelerated Solvent Extraction

A Speed Extractor (E-916/E-914, BUCHI, Flawil, Switzerland) was used for percolation at high pressure. The operating conditions of ASE that were applied for this extraction method were fixed at 100 bar for extraction pressure and 50 °C for temperature for one extraction cycle. There was 1 min of pre-heating time before 5 min of extraction and finally 3 min of discharge. Then, 6 g of olive pomace powder was mixed with sand and filled into extraction cells of 120 mL, and then, approximately 60 mL of solvent (EtOH/H_2_O 60:40 *v*/*v*) was injected into the cells to prompt the extracts into the collection vials.

#### 2.4.2. Ultrasonic Assisted Extraction Probe

The UAE of olive pomace was performed using an ultrasonic probe (20 KHz, 1 kW, UIP 1000 hdT, Hielscher Ultrasonic GmbH, Teltow, Germany). Fifteen grams of olive pomace dried powder was immersed in 150 mL of hydroalcoholic mixture (EtOH/H_2_O 60:40 *v*/*v*). The solution was introduced in a double jacket reactor connected to a cooling/heating system (Ministat 125, Huber, Berchin, Germany) to maintain the temperature at 50 °C during the extraction. Afterwards, this mixture was submitted to ultrasound for 5 min with ultrasonic power of 130 W.

#### 2.4.3. Ultrasonic Assisted Extraction Bath

The UAE was conducted in an ultrasonic extraction reactor PEX 1 (REUS, Contes, France) composed of a stainless steel jug with dimensions of 14 × 10 cm and a maximum capacity of 1 L, equipped with a transducer at the base of the jug operating at a frequency of 25 kHz, with a maximum input power (output power of the generator) of 350 W. For the control and regulation of the extraction temperature of 50 °C, a double-layered mantle provided water circulation with a cooling/heating system (Ministat 125, Huber, Berchin, Germany). Then, 15 g of olive pomace dried powder, immersed in 150 mL of extraction solvent (EtOH/H_2_O 60:40 *v*/*v*), was introduced into the ultrasonic reactor. During the 5 min of extraction, the solution was kept under stirring. The temperature was previously set at 50 °C in the device and the solution was kept under stirring during 5 min of extraction.

#### 2.4.4. Microwave Assisted Extraction

A Milestone EOS-GR (Microwave gravity system, Milan, Italy) system equipped with an infrared temperature sensor and two magnetrons of 950 W was used for this MAE. In a round bottomed flask of 500 mL, 15 g of powder of dried olive pomace was immersed in 150 mL of ethanol/water mixture (60:40 *v*/*v*). The power was previously fixed at 100 W and the temperature was measured just after the 5 min of extraction (between 52 °C and 54 °C).

#### 2.4.5. Bead Milling Extraction

BM was carried out in an ULTRA-TURRAX tube drive (UTTD, Ika, Staufen, Germany). In a drive tube of 20 mL, 1 g of olive pomace mixed with 20 g of ceramic beads and with 10 mL of pre-heated ethanol/water mixture (60:40 *v*/*v*) were processed at 4000 rpm for 5 min. The extracts were filtered and stored at −18 °C until analysis.

### 2.5. Analytical Procedures

#### 2.5.1. Determination of Total Phenolic Content (TPC)

The determination of total phenolic content in the extracts previously diluted at 1:1000 was carried out using the Folin–Ciocalteu test [28]. Briefly, a 2.5 mL portion of diluted extract and 125 µL of Folin–Ciocalteu reagent were mixed together; after 3 min, 50 µL of saturated solution of aqueous sodium carbonate was added. The whole was mixed in a test tube and allowed to equilibrate at room temperature in the dark for 1 h, then the absorbance was read spectrophotometrically at 750 nm (UV-Visible specord S600, Analytic Jena, Jena, Germany). The total phenolic concentration was calculated using tyrosol for the calibration curve, and then results were expressed as milligrams of tyrosol equivalent (TE) per gram of dried olive pomace powder (DOP). All experiments were performed in triplicate.

#### 2.5.2. UPLC Analysis

The quantification of total phenolic content was carried out with an Acquity UPLC^®^ H-Class plus system (Waters, Milford, MA, USA) equipped with a diode array detector (DAD 200–800 nm, Waters, Milford, MA, USA). For the determination of the phenolic content in all the extracts, a reversed phase UPLC method for phenolic compounds in olive pomace was applied as described by Malapert et al. [27]. The separation was performed on a column Acquity UPLC-BEH C18 (2.1 mm × 50 mm, 1.7 µm particle size). The mobile phase was water acidified at 1% by formic acid (Solvent A) and acetonitrile (solvent B). The gradient was linear and the proportions of solvent B used were as follows: 0–10 min 1–20%, 10–12 min 20–30%, and 12–14 min 30–100%. The injection volume was 1 μL, and the column temperature was kept at 35 °C. Along the three steps of the gradient, the flow rate was 0.30, 0.35, and 0.40 mL/min. The spectroscopic detection was performed between 200 and 600 nm with 1.2 nm of resolution. The quantification of the main phenolic compounds was carried out from calibration curves constructed with hydroxytyrosol, tyrosol, caffeic acid and *p-*coumaric acid. Phenolic compounds were detected and quantified at 280 nm. All analyses were made in triplicate.

### 2.6. Statistical Analysis

The expression of continuous variables was taken as mean value ± standard deviation (SD). The normality distribution of all quantitative variables (TPC, 3-hydroxytyrosol, tyrosol, caffeic acid and *p*-coumaric acid) was analysed and validated using the Shapiro–Wilk test. Then, in order to compare mean of TPC between all solvent ratio groups at 20 °C and 50 °C, we used a parametric test ANOVA and a post-hoc test (Tukey).

The Student test was used to compare the mean value of the quantity of 3-hydroxytyrosol, tyrosol, caffeic acid and *p*-coumaric acid after 20 min maceration between the two temperature conditions of 20 °C and 50 °C. Statistical analyses were carried out using IBM SPSS Statistics 23 software (Armonk, NY, USA) at a 95% confidence level (*p* value < 0.05 that was considered statistically significant). All experiments were carried out in triplicate.

## 3. Results and Discussion

### 3.1. Theoretical Solubilities: COSMO-RS Calculations

The evaluation of the solubility capacity of extraction solvents toward targeted compounds in the matrix is a crucial step for extraction. In this context, the COSMO-RS software was used in this work as a powerful tool to compare different EtOH/H_2_O mixtures in order to determine the most efficient ratio to solubilize the main phenolic compounds in olive pomace. To describe the local surface polarity, COSMO-RS calculations present the conductor–polarization charge density (σ) on each molecule’s surface. Afterwards, the description of differences and similarities between the ethanol/water mixture and the investigated polyphenols was analyzed in terms of the σ-profiles, the σ-potentials and σ-surfaces of each compound. In this way, the relative solubilities of the targeted compounds (solutes) in the solvent system were predicted. The calculations were performed in mass:mass with the consideration of investigated polyphenols as solid solutes.

**Figure 3:**
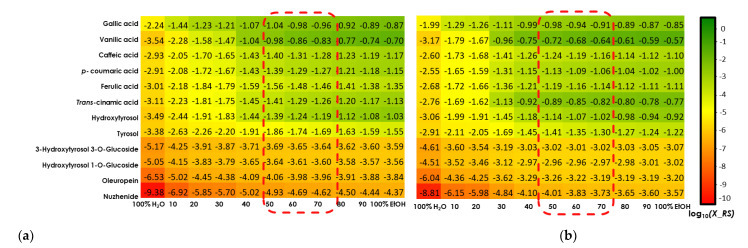
COSMO-RS solubility prediction at 20 °C (**a**) and at 50 °C (**b**) with several ratios of EtOH/H_2_O. Green color: high probability of solubility (≥60%). Yellow color: medium probability of solubility (20–60%). Red color: low probability of solubility (<20%).

Figure 3a,b (20 °C and 50 °C) show the relative solubility values (log10(x_RS)) of investigated polyphenols in different ethanol/water mixtures. The best extraction solvent for the majority of solutes was found to be 100% of ethanol, which is therefore taken by the software as a reference; it then gives all the other solvents relative to it. The different colors, shown in the table of COSMO-RS results, provide information on the relative solubility of phenolic compounds in the solvent mixture. Green values represent a higher solubility index (0 to −1) compared to the other EtOH/H_2_O ratios in the solvent system, yellow values stipulate a medium solubility index (−1 to −4) relative to that of the reference solvent, while red values indicate a very low solubility index (<−4). According to Figure 3a,b, it can be noticed that the solubility index of all investigated phenolic compounds is improved by increasing the temperature. Indeed, the range of green values was extended to (30:70) of EtOH/H_2_O at 50 °C (Figure 3b) compared to that at 20 °C (40:60 of EtOH/H_2_O) (Figure 3a).

It can be clearly observed that all six acids, as well as *3-*hydroxytyrosol and tyrosol, have a high solubility index to the ratio 50:50 ethanol/water for both temperatures. Gallic acid also shows high solubility for EtOH/H_2_O ratios (40:60, 30:70 and 20:80), and vanillic acid and *trans*-cinnamic acid for EtOH/H_2_O ratios (40:60 and 30:70). On the other hand, both glycosidic forms of hydroxytyrosol present a medium solubility index in the reference solvent and it decreases through the solvent system to have mostly a low solubility in 100% water. Nuzhenide followed by Oleuropein shows the lowest solubility values even in 100% EtOH, as indicated by its medium solubility index.

### 3.2. Maceration

**Figure 4:**
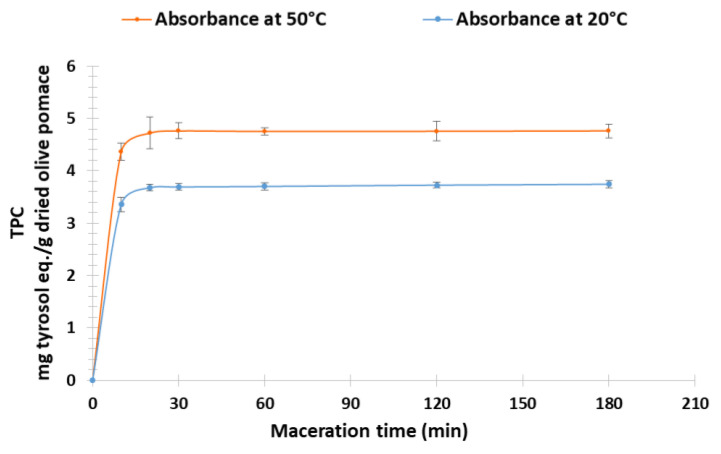
Kinetic of dried olive pomace extraction at 20 °C and at 50 °C.

To determine the optimal extraction conditions, the extraction duration was evaluated as a function of TPC obtained by CM with EtOH/H_2_O ratio (60:40) as solvent for temperatures of 20 °C and 50 °C. Kinetic monitoring shows similar profiles (Figure 4). However, the comparison of extraction yields shows that the temperature influences the yields according to the prediction of COSMO-RS. The maximum yields obtained are 3.75 ± 0.07 mg TE/g DOP at 20 °C and 4.76 ± 0.13 mg TE/g DOP at 50 °C after 20 min of maceration.

**Figure 5:**
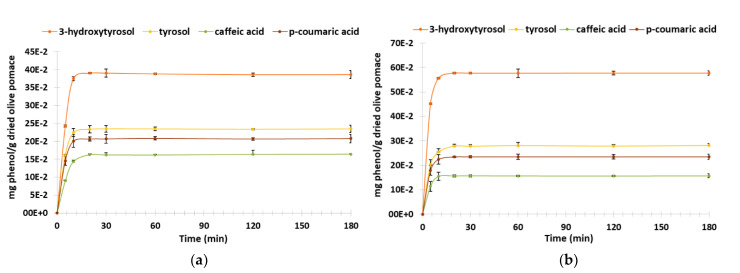
Kinetic of dried olive pomace extraction at 20 °C and at 50 °C: UPLC 20 °C (**a**), UPLC 50 °C (**b**).

UPLC analyses corroborate the TPC results for the four quantified phenols: the highest extraction yields were obtained at 50 °C. The highest concentration was obtained for *3-*hydroxytyrosol with (39.0 ± 1.9) 10^−2^ and (57.6 ± 1.2) 10^−2^ mg HT/g DOP at 20 °C and 50 °C, respectively, followed by tyrosol with (23.4 ± 1.0) 10^−2^ and (27.9 ± 0.7) 10^−2^ mg TY/g DOP at 20 °C and 50 °C, respectively; then, *p*-coumaric acid with (20.6 ± 0.5) 10^−2^ and (23.5 ± 0.5) 10^−2^ mg *p*-CA/g DOP at 20 °C and 50 °C, respectively, and then caffeic acid with (16.3 ± 0.5) 10^−2^ mg CA/g DOP at both temperatures (Figure 5).

The comparative analysis using the Student’s test of the phenolic compounds’ extraction mean rates between two temperature conditions (20 °C vs. 50 °C after 20 min of maceration) show a statistical difference for 3-hydroxytyrosol, tyrosol and *p-*coumaric acid. However, concerning caffeic acid, there is no significant difference of mean rate between 20 °C and 50 °C (*p*-value = 0.11) (Table 2).

These statistical results allowed us to prove the increased effect of extraction rate related to 3-hydroxytyrosol, tyrosol and *p*-coumaric acid at 50 °C.

**Figure 6:**
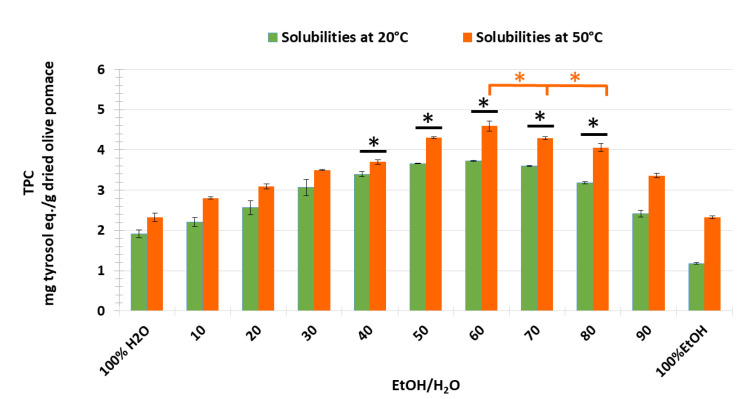
TPC extracted from olive pomace by CM with variation of EtOH/H_2_O ratio from 0 to 100% at 20 °C and at 50 °C; *: p ≤ 0.05.

We can conclude that the extraction yield of most of the phenolic compounds was improved by increasing the temperature, except that of caffeic acid, which was not affected by temperature. The extraction duration evaluated as a function of TPC obtained by CM with EtOH/H_2_O ratio (60:40) as solvent for the temperatures of 20 °C and 50 °C was then applied to study all the different EtOH/H_2_O ratios that were studied previously with the COSMO-RS software. Experimental results showed that extracts obtained with (60:40) of EtOH/H_2_O had the highest TPC values with 3.73 ± 0.02 at 20 °C and 4.59 ± 0.12 mg TE/g DOP at 50 °C, followed by those of (70:40) and (50:50) of EtOH/H_2_O with 3.60 ± 0.02 and 3.66 ± 0.09 at 20 °C, and 4.29 ± 0.03 and 4.30 ± 0.02 mg TE/g DOP at 50 °C, respectively (Figure 6). Moreover, the results of ANOVA analysis to compare mean groups (from 40:60 to 80:20 EtOH/H_2_O) at two different extraction temperature conditions show the presence of statistical differences between each percentage group (*p value._ANOVA_ < 0.05*). In addition, using a post-hoc test, the TPC extraction rate related to the solvent ratio of 60:40 EtOH/H_2_O at 50 °C was found to be statistically different from all other rates at both temperatures (Tukey-*p value < 0.05*). On the contrary, the extraction rate obtained with the same solvent ratio at 20 °C was not found to be statistically different from those determined using 50:50 and 70:30 EtOH/H_2_O at 20 °C, as well as using 40:60 EtOH/H_2_O at 50 °C. In addition, the extraction rates of TPC corresponding to 50:60 and 70:30 EtOH/H_2_O are not statistically different at 50 °C. This leads to the conclusion that the ratio 60:40 EtOH/H_2_O at 50 °C could be the most efficient solvent for extracting the majority of phenolic compounds from olive pomace (Figure 6).

The lowest values were found when olive pomace was extracted with 100% EtOH or 100% water at both temperatures, which is contrary to the COSMO-RS prediction. The contradiction with COSMO-RS solubility prediction for the higher percentage of ethanol can be explained by the fact that the COSMO-RS calculation of the solubility index of solutes in the solvent system follows a linear trend, i.e., the increase in solubility values results from the increase in ethanol concentrations.

Hence, it can be concluded that the threshold for a valid COSMO-RS prediction model was considered in a solvent system with half water and half ethanol or 60% of ethanol. Consequently, this prediction model can only be validated by the experimental results up to a ratio of 60% ethanol. From 70% ethanol, the decrease in the solutes’ solubility can be explained by the change in polarity of the solvent system, which is not favorable for efficient solubilization [15,29].

**Figure 7:**
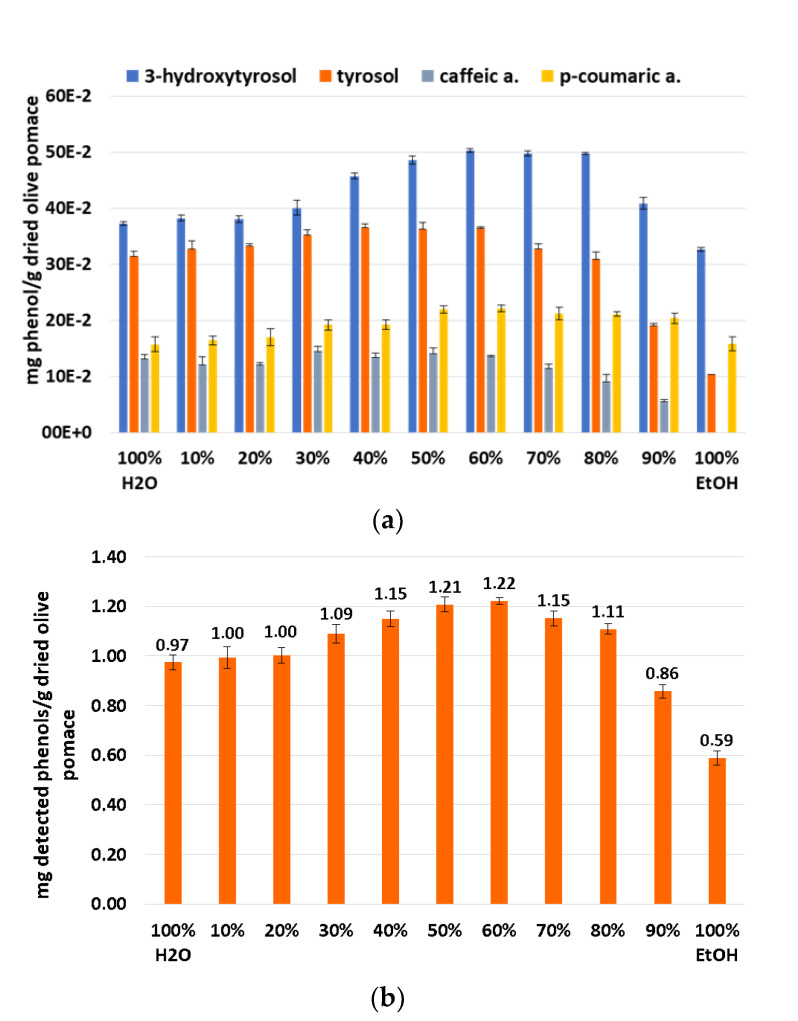
Determination of experimental solubilities at 50 °C of different ratios EtOH/H_2_O by UPLC, quantified phenols (**a**) and sum of quantified phenols (**b**).

After this first screening with the Folin–Ciocalteu spectrophotometric test, a second screening by UPLC/DAD was performed under the same conditions but only at 50 °C. As can be seen in Figure 7a, 3-hydroxytyrsol and p-coumaric acid follow the same trend as the TPC results while tyrosol and caffeic acid exhibit different behavior depending on the solvent ratios. The sum of these quantified phenols shown in Figure 7b follows the same trend as that presented by the TPC results. This also corroborates that the 60:40 EtOH/H_2_O mixture provides the best solubility for the target phenolic compounds followed by the 50:50 ethanol/water mixture.

Based on the experimental results that correlated well with the COSMO-RS prediction, the best EtOH/H_2_O mixture that was selected is (60:40). These results have already been reported in the literature for the extraction of phenolic compounds from olive pomace [15]. Furthermore, the extraction temperature was selected as 50 °C, which presents more efficient results than at 20 °C.

### 3.3. Intensification

Different innovative green extraction techniques were performed and compared to CM to improve the yield and reduce the extraction time. Therefore, olive pomace powder was dissolved in an EtOH/H_2_O mixture (60:40) with a sample-to-solvent ratio equal to 1:10 (g/mL) and was subjected to different eco-extraction methods for 5 min at 50 °C: ASE, UAE-P, UAE-B, BM and MAE.

**Figure 8:**
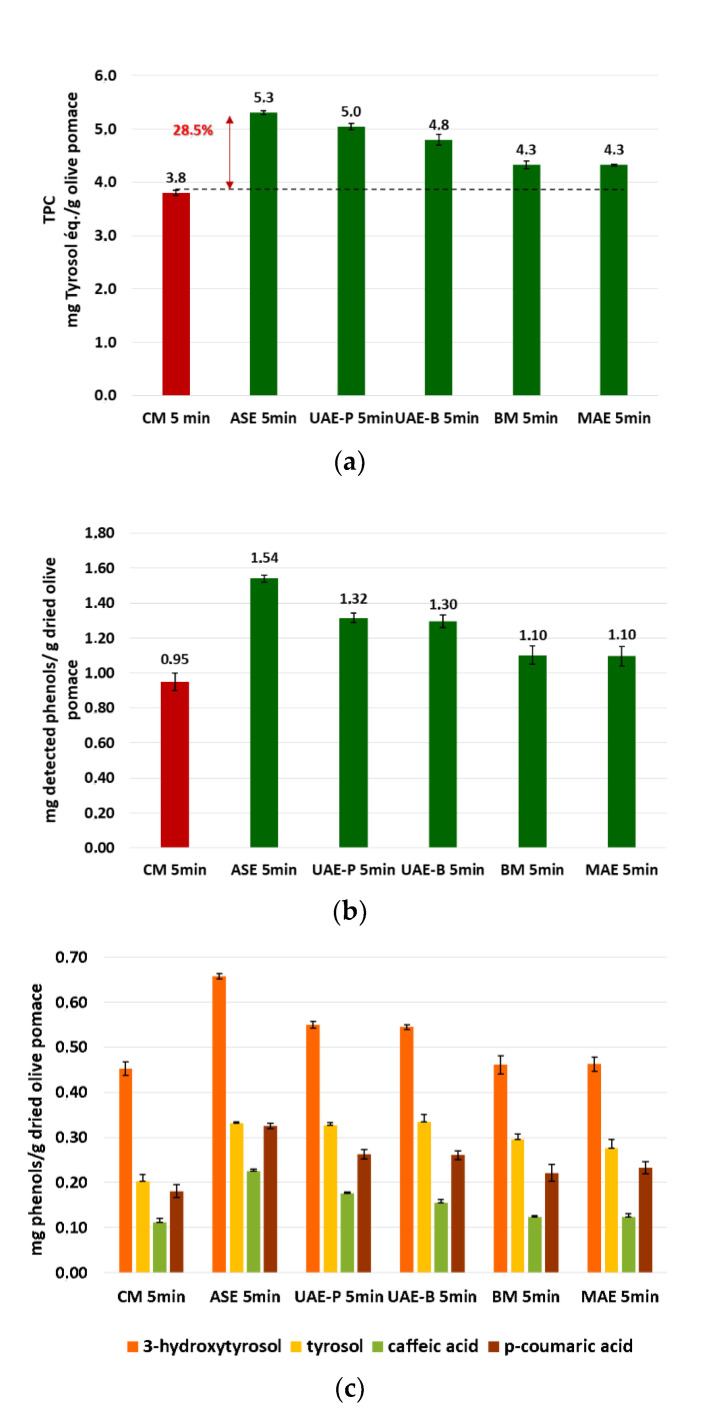
Comparison of the green extraction techniques by TPC determination (**a**), sum of quantified phenols by UPLC (**b**), and quantified phenols by UPLC (**c**).

Figure 8a shows that the phenolic extract recovered using ASE presents the highest TPC value (5.31 ± 0.04 mg TE/g DOP), followed by UAE, which reveals higher levels of the probe system (5.04 ± 0.06 mg TE/g DOP) compared to the bath system (4.80 ± 0.11 mg TE/g DOP). The lowest extraction yields correspond to BM (4.32 ± 0.70 mg TE/g DOP) and MAE techniques (4.33 ± 0.02 mg TE/g DOP) with almost identical results (Figure 8a).

**Figure 9:**
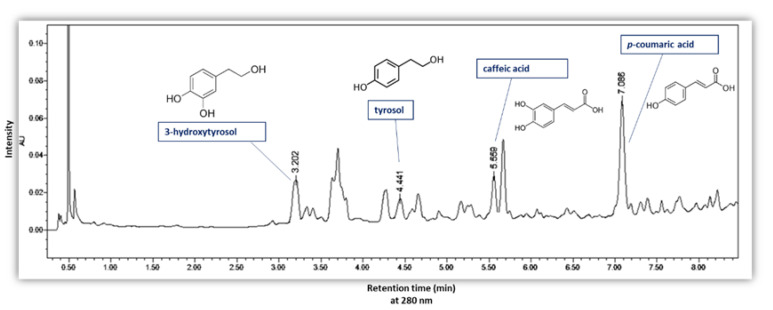
UPLC chromatogram of phenolic compounds from olive pomace powder extracted by ASE with detection at 280 nm.

The results of TPC in the different extracts were perfectly confirmed by UPLC analysis (Figure 8b,c). In Figure 9, a**n** UPLC chromatogram of phenolic compounds from olive pomace powder extracted by ASE is presented. Figure 8b presents the sum of the four phenolic compounds quantified by UPLC, detailed in Figure 8c. The results show that the sum of phenols detected by UPLC follows exactly the same trend as TPC. As shown in Figure 8c, *3-*hydroxytyrosol has the highest concentration, ranging from (45.2 ± 1.4) 10^−2^ to (65.7 ± 0.6) 10^−2^ mg HT/g DOP, followed by tyrosol with (20.3 ± 1.4) 10^−2^ to (33.2 ± 0.2) 10^−2^ mg TY/ g DOP and *p-*coumaric acid with values between (18.0 ± 1.4) 10^−2^ and (32.5 ± 0.6) 10^−2^ mg p-CA/g DOP. The lowest concentration was detected for caffeic acid as (12.0 ± 0.8) 10^−2^ to (22.4 ± 0.5) 10^−2^ mg CA/g DOP.

ASE increased the extraction yield by 28.5% compared to CM for a duration of 5 min. The ultrasonic techniques also showed significant improvement in extraction efficiency, considering that the ultrasonic probe is a little more efficient than the bath one.

Extraction techniques based on BM and MAE were found to be the least efficient investigated techniques, with extraction yields slightly higher than the results obtained by CM.

To conclude, ASE was found to be effective as an innovative technique to enhance the extraction of phenolic compounds from dried olive pomace powder. The efficiency of the ASE method would be explained by the effect of the pressure applied during extraction. This latter allows the improvement of the diffusivity of the EtOH/H_2_O mixture through the pores of olive pomace particles. Others studies have also shown significant results with ASE for the extraction of phenolic compounds [16,21,30].

### 3.4. Comprehension of Mechanism

**Figure 10:**
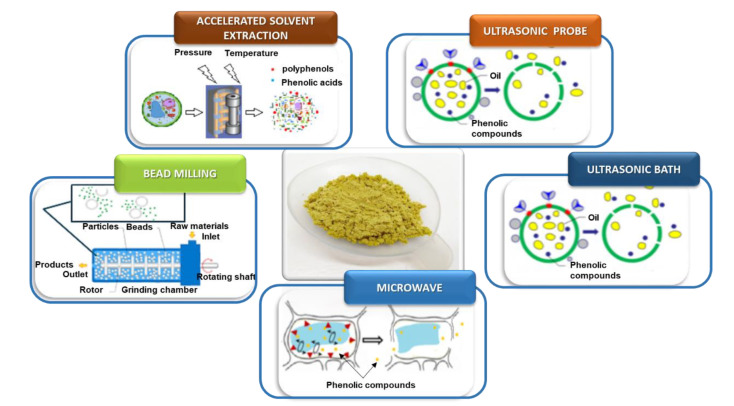
Effect of extraction processes on the structure of dried olive pomace powder.

During the extraction process (Figure 10), the matrix effect depends on its physical and chemical composition. Solid, granular or powdered compounds can differ significantly, which generates differences in the extraction process. Additionally, the large number of organic compounds present in the matrix increases the complexity of extraction procedure. The type and properties of the solvent, such as polarity or viscosity, also have an important role in the extraction phenomenon.

ASE turned out to be the best technique for the extraction intensification of phenolic compounds from dried olive pomace powder. Furthermore, the combination of high pressure and temperature improves performance due to the disruption of surface equilibrium and the resulting solubility and mass transfer effects. Using the ASE technique, the powder of the olive pomace sample is enclosed in a cartridge filled with the hydroalcoholic solvent and then extracted statically under a pressure of 100 Bar and a temperature of 50 °C for only 5 min. After that, the sample extract is purged from the cell into the collection vessel using compressed gas.

A temperature of 50 °C disrupts the solute–matrix interaction and lowers the surface tension of the matrix, solvent and solutes, which favorizes the diffusion phenomenon. On the other hand, the high temperature decreases the viscosity of solvent leading to an improvement in its ability to solubilize targeted compounds from the matrix [31]. From the pressure point of view, the high pressure used facilitates the extraction of phenolic compounds trapped in matrix pores. Indeed, the pressure forces the solvent into the areas of matrices that are not normally accessible by the solvent under atmospheric conditions.

We found that, in the case of the UAE technique, the use of probe sonication produces better results than the bath. These results are in full agreement with the findings of Jamalabadi et al. (2019) [32], who showed that probe sonication was more efficient than the bath one in terms of influencing the physicochemical and microstructural properties of matrixes, which leads to the intensification of extraction yields.

On the other hand, it seems that the MAE and BM techniques have almost the same effect on the structure of the studied matrix. The dried olive pomace, in its powder form, probably cannot be influenced by the movement of the beads, which normally leads to the destruction of the grains in a smaller size increasing the contact surface between solute and solvent and thus improving the extraction of phenolic compounds [33].

For MAE, the lack of stirring is not favorable for improving the solubility of phenolic compounds in the solvent. The microwave irradiation does not stimulate the cells, which typically leads to its disintegration, and then promotes the release of targeted molecules [24].

## 4. Conclusions

The objective of this study was to enhance the extraction yield of phenolic compounds from olive pomace powder “Aglandau olives variety” by reducing the time of manipulation while working in a sustainable and environmentally friendly manner. It was established that the optimal extraction yield was achieved using a solvent ratio of (EtOH/H_2_O) (60:40 *v*/*v*), under 50 °C and within 20 min. These optimized experimental conditions were validated via statistical methods. Furthermore, results showed that ASE was the most effective method for extracting phenolic compounds from dried olive pomace powder (5.3 milligrams of tyrosol equivalent (TE) per gram of dried olive pomace powder (DOP)) compared to CM (3.8 mg TE/g DOP). It is well known that olive pomace biophenols are endowed with powerful biological activities. Hence, this by-product would be an interesting source for high added-value phenolic compounds that are useful as alternatives for the undesirable chemical additives in various cosmetic, agro-food and pharmaceutical preparations.

## Figures and Tables

**Table 1:** Summary table of some articles using ethanol/water as extraction solvent.

Plant Matrix	Components of Interest	Optimum Conditions	Process	Observations	Ref.
**Common sage**	rosmarinic acid, carnosic acid	55–75% EtOH T°: 40 °C Solvent-to-sage ratio 6:1 (*v*/*w*)	CM	The highest rate of phenolics obtained was 6.9% of rosmarinic acid and 10.6% carnosic acid).	[14]
**Olive pomace**	hydroxytyrosol, tyrosol and oleuropein	60% EtOH T°: 70 °C solvent to sample ratio 5:1 (*v*/*w*).	CM	The use of 60% ethanol (instead of 40%) slightly increased the TPC values (3.62 mg GAE/g).	[15]
**Sorghum brans**	phenolic compounds	50 and 70% EtOH T°: 120 °C and 150 °C: 120 °C and 150 °C	ASE	The total phenols at 150 °C were similar to those obtained at 120 °C when aqueous ethanol (50 and 70%) was used (45 mg GAE/g).	[16]
**Mango seeds**	gallic acid, caffeic acid, rutin	50% EtOH T°: ambiant	CM	The ethanol–water binary solvent system had the highest TPC (101.68 mg GAE/g).	[17]
**Grape seeds**	phenolic compounds	50% EtOH solvent to sample ratio 5:1 (*v*/*w*)	CM	Under these conditions, the maximum recovery of polyphenols was 11.4% of the total weight of grape seeds.	[18]
**Wheat bran**	phenolic compounds	64% EtOH T°: 60 °C 25 min of extraction	UAE	The highest amounts of phenolic content (3.12 mg GAE/g) can be recovered under these conditions.	[19]
**Grape seeds meal**	gallic acid, catechin, epicatechin, epicatechin gallate and procyanidin	50% EtOH T°: 65 °C double stage extraction liquid to solid ratio of 7.5:1 (*v*/*w*)	CM	These conditions were considered the best procedure for the extraction of phenolics from ground grape seed meal (1866 mg/kg).	[20]
**Wild nettle leaves**	hydroxyl-cinnamic acids, flavonoids	96% EtOH T°: 110 °C 10 min of extraction 3 or 4 cycles	ASE	ASE achieved the highest yields of analyzed compounds with chlorogenic acid (248.49 mg/100 g dm).	[21]
**Fresh olive fruit**	gallic acid, quercetin luteolin, rutin	Water T°: 100 °C	ASE	The best recovery of phenolics was obtained in water (130 mg/g) in a short time (19.5 min).	[22]
**Fresh carrots**	carotenoids	Sunflower oil T: 40 °C solvent to sample ratio 5:1 (*v*/*w*) 20 min of sonication	UAE	In only 20 min, the UAE using sunflower as a solvent achieved the highest yield of β-carotene (334.75 mg/L).	[23]

**Table 2:** A comparative table of the mean values of quantified phenolic compounds at 20 min maceration at 20 °C and 50 °C.

Quantified Phenolic Compound	Mean ± SD at 20 °C	Mean ± SD at 50 °C	*p* Value
3-hydroxytyrosol (10^−2^ mg/g DOP *)	39.0 ± 1.9	57.7 ± 0.3	***P_s_ ≤ 0.001***
Tyrosol (10^−2^ mg/g DOP *)	23.4 ± 1.0	27.9 ± 0.7	***P_s_ = 0.003***
caffeic acid (10^−2^ mg/g DOP *)	16.3 ± 0.3	15.6 ± 0.4	*P_s_ = 0.11*
*p*-coumaric acid (10^−2^ mg/g DOP *)	20.6 ± 0.5	23.5 ± 0.3	***P_s_ = 0.001***

* DOP: dried olive pomace powder.

## Data Availability

Not applicable.
